# To charge or not to charge: reducing patient no-show

**DOI:** 10.1186/s13584-023-00575-8

**Published:** 2023-08-08

**Authors:** Gideon Leibner, Shuli Brammli-Greenberg, Joseph Mendlovic, Avi Israeli

**Affiliations:** 1 Faculty of Medicine, Hebrew University-Hadassah, Jerusalem, Israel; 2 Ministry of Health, Jerusalem, Israel; 3 Department of Pediatrics, Shaare Zedek Medical Center, Affiliated With the Hadassah-Hebrew University School of Medicine, Jerusalem, Israel; 4 Dr. Julien Rozan Professor of Family Medicine and Health Care, Faculty of Medicine, Hebrew University-Hadassah, Jerusalem, Israel

**Keywords:** Health policy, Health Management, No-show, Health Economics

## Abstract

**Background:**

In order to reduce patient no-show, the Israeli government is promoting legislation that will allow Health Plans to require a co-payment from patients when reserving an appointment. It is hoped that this will create an incentive for patients to cancel in advance rather than simply not show up. The goal of this policy is to improve patient access to medical care and ensure that healthcare resources are utilized effectively. We explore this phenomenon to support evidence-based decision making on this issue, and to determine whether the proposed legislation is aligned with the findings of previous studies.

**Main body:**

No-show rates vary across countries and healthcare services, with several strategies in place to mitigate the phenomenon. There are three key stakeholders involved: (1) patients, (2) medical staff, and (3) insurers/managed care organizations, each of which is affected differently by no-shows and faces a different set of incentives. The decision whether to impose financial penalties for no-shows should take a number of considerations into account, such as the fine amount, service type, the establishment of an effective fine collection system, the patient’s socioeconomic status, and the potential for exacerbating disparities in healthcare access. The limited research on the impact of fines on no-show rates has produced mixed results. Further investigation is necessary to understand the influence of fine amounts on no-show rates across various healthcare services. Additionally, it is important to evaluate the implications of this proposed legislation on patient behavior, access to healthcare, and potential disparities in access.

**Conclusion:**

It is anticipated that the proposed legislation will have minimal impact on attendance rates. To achieve meaningful change, efforts should focus on enhancing medical service availability and improving the ease with which appointments can be cancelled or alternatively substantial fines should be imposed. Further research is imperative for determining the most effective way to address the issue of patient no-show and to enhance healthcare system efficiency.

## Background

A smoothly operating patient appointment system is crucial to the provision of healthcare; the phenomenon of no-shows (namely, when a patient fails to attend a scheduled appointment/treatment without prior notice) represents a significant challenge to that system, by wasting the time and resources of healthcare providers and therefore degrading patient health and well-being.

To reduce the occurrence of no-shows, the Israeli government promoted legislation along an expedited track that would authorize Health Plans (HPs) to collect a co-payment from patients when they make an appointment and would require them to remind patients about their scheduled appointment 24 h beforehand. Patients would still be able to cancel appointments, but if they fail to show up without having cancelled then the HPs would not be obligated to provide a refund [1]. Consequently, this system would create an economic incentive for patients to cancel in advance and will reduce the number of no-shows. However, due to the complex and sensitive nature of the proposed legislation, and concerns regarding potentially adverse effects on vulnerable populations, it was decided that the passage of the legislation would not be expedited but instead would follow the normal legislative route.

According to the definition provided by the Merriam-Webster Dictionary [2], a fine is a “sum imposed as punishment for an offense”. The proposed legislation, however, will not truly impose a fine on patients. Rather, it shifts the collection of the co-payment for medical services to the time of making the appointment and makes the payment non-refundable in the case of a no-show. Although this technically differs from a traditional monetary fine, it essentially serves as a monetary penalty for a no-show. Therefore, for the purposes of the analysis, we will indeed view this payment as a monetary fine.

The goal of such a policy is to improve patient access to medical care and ensure that healthcare resources are utilized effectively. In this study, we intend to explore this phenomenon to support an evidence-based decision on the legislation’s desirability, and to determine whether the findings of previous studies can shed light in this direction.

The paper proceeds as follows: in the first part, we discusses the no-show phenomenon in Israel and other countries. The second section focuses on the various stakeholders and how no-show affects each of them. In the third section we discuss the economics of reducing no-show, and the approach to economic analysis of the various interventions. Finally, we will discuss the use of financial sanctions to reduce no-shows.

## Main text

### The no-show phenomenon

There has been a great deal of research on no-shows in Israel and many other countries. No-shows prevalence exhibits variation across countries and medical specialties, however, the reported rates are subject to the criteria used to define a no-show occurrence. Specifically, the definition may encompass not only the absence of a patient without prior notification but also instances of late cancellations. Furthermore, the reported rates may pertain to all appointments or only to initial visits [3].

No-show rates vary by location and by the medical service subspecialty. For example, in a systematic review of 105 papers, average no-show rates were 23%, with African studies showing the highest rate (43.0%) and those in Europe and Oceania showing the lowest (19.3% and 13.2%, respectively). In the case of medical specialties, the lowest median no-show rate (11.2%) was found in “other” health services (i.e., pulmonary tuberculosis, intravenous therapy, rheumatology, hand surgery, urology, ophthalmology, obstetrics/gynecology, and oncology) and the second lowest (14.6%) in medical examinations; the highest rates were observed in psychiatric care (57.3%) followed by endocrinology (36.0%) [3].

An Israeli study carried out in Shaare Zedek Medical Center found that 15% of ENT appointments and 16% of orthopedics appointments did not take place due to patient no-show [4]. Another study, which examined reasons for no-show in child development centers, observed an average rate of 26.6%, with the most common reason being an unexpected event (26.0%), followed by problems in obtaining a financial commitment from the HP that is paying for the treatment (23.4%) [5].

Various methods are used to reduce no-show rates, including reminders via email, Short Message Service (SMS) [6, 7], or phone calls, whether automated or manual [7]; upfront payments; no-show fines [8, 9]; and other sanctions [10]. There is variation in the effectiveness of each on the no-show rate. As part of the effort to reduce no-shows, models have been developed in an attempt to identify the patients with a high risk of no-show [11, 12], which makes it possible to target the interventions.

### The stakeholders

The cost of no-shows can be viewed from various perspectives. Bech defines two main no-show costs [13]: “Non-attendance gives rise to two types of costs: social costs and financial costs to providers. The social costs of non-attendance are the lost value of the unused or misused resources resulting in lower productivity and lost benefits…. The financial cost is the providers’ loss of income caused by non-attendance*.*” These costs were discussed briefly in the previous section. We propose that a more effective way to differentiate the costs of no-show is according to the various stakeholders. This is especially true for the providers whom he mentions, that, in our opinion, at least in certain health systems should be separated to medical staff and insures or managed care organizations (for the purpose of this paper, the term “managed care organization” will henceforth encompass both conventional healthcare providers and insurers that function as primary healthcare providers within specific healthcare systems). Therefore, in the context of no-show, there are three main stakeholders that we will refer to: patients, medical Staff (e.g., physicians, nurses, technicians etc.), and managed care organizations (e.g., hospital, HP, health institutions, etc.). The interactions between them are complex and our goal is to disentangle the mutual effects in the context of no-shows.

#### Patients

From the patient’s perspective, the prime objective of a medical appointment is to obtain health services, which include diagnosis, consultation and medical treatment. No-shows pose a threat to a patient’s health and it can be used as an indicator for patients who are at increased risk. No-shows interfere with the patient's therapeutic and diagnostic sequence, such as chronic disease control [14] and radiological screening tests for early detection of diseases [15], which in turn may lead to poor individual health outcomes. One study found missed appointments to be a strong predictor of diabetic status (as measured by HbA1C) and that there is a 1.24-fold increase in the risk of poor health for every 10% increment in the missed appointment rate [16]. Another study found no-show patients to have higher rates of emergency department (ED) [17] visits and hospital admissions [18]. Nonetheless, causality between the two is hard to prove, and it is possible that no-shows are an indicator of poor health status or poor adherence to medical protocols.

No-shows also have indirect effects on patients, by way of reduced availability of medical services and longer waiting times. A no-show is an occupied slot in the schedule, making it more difficult for other patients in need of medical care to access that specific service. To deal with this phenomenon, managed care organizations often overbook and optimize their schedule, which can lead to unpredictability in patient flow and scheduling conflicts. In some cases, this results in a larger number of patients eventually attending their appointments, resulting in longer clinic waiting times and shorter patient-physician interactions. These effects can at the very least lead to inconvenience for patients, and may negatively impact the quality of care provided, or in the worst case may lead to patients refraining from seeking medical care.

#### Medical staff

The implications of no-shows for medical staff are more complex and to a large extent are determined by their wage agreement. If the medical staff are compensated on a fee-for-service basis or according to a fixed amount per appointment, any unfilled time slot results in a loss of income. Same-day appointments, whether walk-ins, waiting lists, or overbooking, may lower the potential losses; however, these are unpredictable and there will still likely be unfilled time slots. In a study conducted on a family healthcare center, which had a 31.1% no-show and cancellation rate, approximately 61.0% of missed or canceled appointments were filled with same-day appointments, while 12.1% of the appointments nonetheless remained unfilled [19].

If the medical staff are paid on the basis of a “fixed” salary, then no-shows have the opposite effect, in that they in fact lighten the workload and allow them to allocate more time to each patient without compromising their income. However, there may be additional consequences to be taken into account (apart from the organizational impact discussed in the next section). First, the long schedules may lead to the inefficient use of the medical staff's time, and may prevent them from devoting time to other tasks. Second, a large number of no-shows may result in inactivity, frustration and anxiety which may affect the medical staff’s well-being. As a result, they may end up being less patient and empathetic toward their patients.

#### Managed care organizations

In the context of managed care organizations, which represent the perspective of the health system, no-shows deny access to medical services for other patients, and there is a direct impact on the availability of appointments and waiting times for medical services. Furthermore, managed care organizations, whether for-profit or non-profit, make extensive investments in infrastructure, including buildings, clinics, medical equipment, etc., and a no-show results in the non-utilization of that infrastructure and the consequent loss of potential revenue.

A study in the US estimated the cost of 146,358 no-shows (a rate of 14.2%) across all clinics in a single medical center in 2008. They calculated a loss of $196 per no-show appointment which amounts to a marginal cost of $28.66 million [20]. Another study examined a family practice center which saw an average of 155 patients daily. It estimated that same-day appointments generate $6.74 less potential income per appointment relative to scheduled appointments, resulting in a loss of $1412.03 per day ($353,008 per year) in potential revenue [19].

The no-show phenomenon creates work for managed care organizations, due to the need to constantly adjust the schedule, overbook, accept walk-ins, reschedule missed appointments, etc. or through the development and implementation of a variety of measures to reduce no-shows, all of which require the investment of costly resources, such as manpower, or the use of costly technologies. These costs need to be taken account in evaluating the effectiveness of interventions.

This issue of no-shows has gained increasing attention from public managed care organizations. This is especially the case in fee-for-service settings in which case healthcare professionals suffer a loss of income from no-shows. This makes it more difficult for a healthcare system to attract workers, especially in light of the fact that initial salaries are low in this sector.

### The economics of reducing no-shows

In determining the relative effectiveness of the various interventions to reduce no-show rates, it is important to examine their cost-effectiveness from the perspective of the health system as a whole, while also taking into account all of the stakeholders.

The most important characteristic of an intervention is the extent to which it reduces no-show rates. No-show rates vary significantly across types of medical service and across the populations being served. Therefore, conclusions regarding the effect should be made in context with the type of service examined. Same-day appointments which partly offset the cost of no-shows also need to be taken into account when calculating the potential loss of income due to no-shows.

The second factor to consider is the costs of implementing each intervention. These include the manpower that is responsible for handling same-day appointments, such as making appointments from a waiting list, retrieving medical information, preparing for unplanned tests, etc. Additionally, there is the cost of the technological platform (such as, for example, an automated phone reminder system) all of which should be taken into account.

The third factor to consider is that in some cases there is a justified reason for a no-show. A decision must be made whether to exempt patients from the penalty in such cases. If a decision is made in favor of exemptions, then specific criteria for granting them are required as well as a system for deciding whether they have been met. However, this leads to other questions, such as who has the authority to decide whether an exemption is justified. Should this be solely within the discretion of the managed care organization, or is it imperative to explicitly outline these situations in legal terms. And in either case, will additional manpower, which will involve additional costs, be needed to carry out such an evaluation? Whatever the case, it can be argued that even if there is a justified reason for a no-show, patients have a responsibility to notify the managed care organizations of a cancellation, even on the same day, and no exemptions should be made.

From the provider's point of view, the benefit of an intervention is measured according to its effectiveness in lowering no-shows relative to the intervention’s operational costs. For example, one study reported on a community health center that introduced a no-show policy, in which patients who consistently miss appointments are barred from receiving treatment, until they are reinstated by means of a formal process. The policy lowered the no-show rate from 34 to 11% within two years. However, an investment of 20 man-hours per week was required to run the system [10]. Another study estimated the cost of a reminder system based on phone and text messages to be 0.41€ per patient on average, with phone reminders being more costly than SMS reminders (0.91€ vs. 0.14€ per patient) [7].

The (social) costs for patients are more complex to measure. One component of these costs is the waiting time for medical services and the medical harm they lead to, which includes, among other things: delays in diagnosis and medical treatment, shortening of appointment duration which may reduce the quality of service, greater disparity in access to health services, etc. Longer clinic waiting times (caused by overbooking) shift the costs from the healthcare provider to the patient who must now wait longer for an appointment [13].

Another important consideration is the impact of waiting times on the manpower needed to provide services. By reducing the waste due to no-shows and increasing utilization, more services can be provided using the same resources, thereby increasing efficiency and saving healthcare system costs. For instance, if a clinic has five physicians capable of treating 20 patients daily, then a 20% no-show rate means that the clinic could be providing the same services with only 4 physicians. Although simplistic, this example serves to illustrate the impact of no-shows on resource utilization and efficiency in the healthcare system.

On the other hand, the imposition of monetary fines to reduce no-shows can potentially have adverse effects. Managed care organizations may overly rely on fines as a solution, thus neglecting other effective measures to minimize no-shows and optimize resource utilization. It therefore should consider a mix of the various strategies, and later reevaluate. Moreover, it will be necessary to put monitoring mechanisms in place, and to conduct periodic evaluations of effectiveness and then carry out any necessary modifications. This will ensure that managed care organizations are implementing the optimal mix of interventions.

### Navigating no-shows: the role of financial sanctions

A number of studies have examined the effect of economic incentives on health behavior [21–24]. In our context, the goal of imposing a fine is to create a financial incentive that will prevent the patients from not showing up to an appointment.

In what follows, we analyze two types of no-show financial sanctions: a self-conditional up-front co-payment paid prior to the appointment and which is non-refundable in the case of no-show; and the imposition of a fine following a no-show. In the first case, the payment is made when an appointment is made, making the collection process easier for the health organization. However, this may lead to the patient forgetting about the financial sanction by the time the appointment arrives, and therefore effective implementation of this method also requires timely reminders of the consequences of a no-show. In the second case, the patient “feels” the fine at the time of the no-show; however, this method requires a legal and bureaucratic infrastructure that will enable managed care organizations to collect the fine.

If we assume that a patient’s utility depends on his income and the benefit from medical services, then his utility from an appointment under the first method of financial sanctions, i.e., a co-payment paid in advance, is described by Eq. (1):

1 $$U_{ m,I} = U_{m} - U_{Ic} - U_{Im}$$

where *U*_*m*_ is the patient’s utility from the medical service *m*, *U*_*Ic*_ is his utility from the income-equivalent of the co-payment amount and *U*_*Im*_ is the utility from the income-equivalent of the costs related to the medical service (such as transportation, loss of a workday, and time spent). Assuming that I_c_ << I_m_ (which is the case in the Israeli health care system^1^) the patient will show up to the appointment only if $U_{m} > U_{Im}$, namely if the utility from the visit is larger than the cost of showing up to the appointment.

In the case of the second method of financial sanction (i.e. the imposition of a fine after a no-show), the patient’s utility is described by Eq. (2) (note that Eq. (2) reduces to Eq. (1) if the patient shows up for his appointment):

2 $$U_{ m,I} = U_{m} - U_{Ic} - U_{Im} - U_{If}$$

where ${U}_{If}$ is the patient’s utility from the income-equivalent of the fine. The assumption in this case is that the patient will be better off showing up to the appointment if ${U}_{m}+{U}_{If}> {U}_{Im}$. In other words, a patient will decide to show up to an appointment only if the benefit of doing so and the potential fine imposed for a no-show outweigh the costs of showing up to the appointment.

The conclusion to be drawn from this simple economic framework is twofold: first, a health system can reduce no-shows by lowering the costs of showing up to an appointment (by increasing accessibility and availability). Second, the more urgent the visit or the more serious the medical case (i.e., U_m_↑), the less the patient will benefit from not showing up, implying that a financial sanction may not be relevant in the case of severely ill patients.

Determining the amount of the fine deserves careful consideration and should be based on an evidence-based approach. As demonstrated above, the amount of the fine is critically important: it should be high enough to deter no-shows, but not overly high given that there is sometimes a valid reason for a no-show. Moreover, imposing fines may alter the utility of other incentives: for example, patients might choose to pay the fine rather than show up to an appointment or cancel it, and the mere imposition of a fine may reduce the moral incentive not to waste the valuable resources of the health system [25–27].

The socioeconomic status of patients should also play a significant role in determining the level of the fine, especially in the case of a fixed fine, which will have a greater impact on lower-income individuals. Therefore, it is important to strike a balance between the effectivity of the fine in modifying patient behavior, and not exacerbating disparities in healthcare provision. An alternative approach is to condition the level of the fine amount on the individual’s income, although implementation of such a system poses challenges due to its inherent complexity and cumbersome nature.

According to the American Medical Association’s Code of Medical Ethics, physicians can charge fees for “missed appointments or appointments not cancelled in advance in keeping with the published policy of the practice”, and they should “clearly notify patients in advance of fees charge” (Opinion 11.3.2) [28]. However, it does not specify the method of calculating the fine. Similarly, the policy of the American Centers for Medicare & Medicaid Services states that physicians can charge for missed appointments as long as this policy applies equally to all patients [29]. Despite these recommendations, no-show fines vary across physicians and across clinics, and there is no centralized database of information on the levels of no-show fines in the US.

In Norway, patients can be charged 1500 NOK (approximately 139€ as of 2023) for a no-show [30, 31]. In a study that mapped the public debate in Norway over the use of a no-show fee, the following considerations were mentioned, among others: (1) there may be factors beyond the patient’s control that lead to no-shows, such as lack of awareness, illness, transportation problems, etc. (2) There may be a negative impact on the patient-physician relationship, due to resentment towards the physicians over the imposition of a fine or against the managed care organization for not finding alternative ways to reduce no-shows, etc. (3) There may be a reduction in equality, in the sense that imposing a fine will have greater impact on lower-income patients, and those with greater medical need. (4) There may be a question regarding the effectiveness of fines in reducing no-show rates [31].

Two studies that examined the effect of imposing a fine found that it reduced no-show rates. In the first, imposing a $30 fine at a mental health outpatient clinic resulted in a reduction in the no-show rate from 20.1 to 9.27% among active patients (12+ sessions over the 18 months of the study) with more than two previous no-shows [8]. The second showed a 14% reduction in no-show rates following the imposition of a fine though it was not statistically significant [9]. However, it is worth mentioning that the studies did not examine the effect of the fine amount. Furthermore, the finding of each study applies to one particular specialty and were conducted in the mid-1990s, with no recent follow-up.

A more recent Danish study used a randomized control experiment consisting of 6746 orthopedic outpatients, with the intervention group informed in advance of a 34€ no-show fine. The results showed no difference in no-show rates between the intervention group and the control group, whose rates were both in the vicinity of 5%. Furthermore, 79% of the 130 fines imposed were unpaid even after two reminder letters [32, 33]. The results may have been due to low no-show rates to begin with in that clinic or that the fine was too low to modify behavior. In short, further experimental studies are needed to clarify the issue.

## Conclusion

In conclusion, the potential adoption of the proposed policy, which includes the introduction of fines or co-payments as a means to address patient no-shows, is contingent upon the government’s decisions and actions. Regardless of the specific policy tool that may be implemented, addressing the no-show phenomenon remains a crucial priority for enhancing health service efficiency and maximize resource utilization. Therefore, a comprehensive examination of a policy’s implementation, including the enforcement of fines or co-payments, and its impact on public health and the healthcare system is called for.

Based on the analysis, it is anticipated that the proposed legislation, which would allow HPs to charge patients a co-payment when making an appointment, will have minimal impact on no-show rates. Financial penalties may create an incentive for patients to cancel in advance, but there is only limited research to confirm this claim. Further research is therefore needed in order to determine the most effective way to reduce no-shows and therefore enhance healthcare system efficiency. Furthermore, strategies to reduce no-shows should be tailored to individual hospitals and specialties, while taking into account patient characteristics and the underlying reasons for no-shows [34]. It is worth noting that regardless of which measure is adopted, there will always be some level of no-shows due to unforeseen circumstances. Therefore, to drive significant change and improve patient access to medical care in conjunction with the implementation of any policy, it is important to improve medical service availability and to streamline the process for appointment rescheduling or cancellation.

Although decision-making in the political arena is inherently different from that in the academic world, should any future proposed legislation be enacted to implement either upfront co-payment or substantial fines, it is imperative to have research-based evidence and recommendations so that legislators can make informed decisions. Only in this way will the legislation take into account the aforementioned challenges and meet the specific needs of the healthcare system and its various stakeholders. Follow-up research should also be conducted in order to evaluate the legislation's effect on no-show rates, its differential effect on various population groups, and its operational costs in the various settings for health service delivery (i.e., HPs, hospitals, and independent clinics), as well as its effect on public health.

Future research should assess the effect of no-shows on physician behavior in terms of clinical practice and the duration of an appointment, and should evaluate the differential impact across types of medical service. Finally, it is important to determine the optimal fine amount that accomplishes the legislator's objectives without compromising public health.

## Data Availability

Data sharing is not applicable to this article as no datasets were generated or analyzed during the current study.

## Abbreviations

HPs: Health Plans
ED: Emergency department

## Footnotes

1 In the current situation, co-payments in Israel are less than $10, and are paid only for the first visit during a quarter.
